# Predicting Sudden Cardiac Death in Heart Failure with Mildly Reduced/Preserved Left Ventricular Ejection Fraction: A Clinical Review

**DOI:** 10.3390/jcm15083041

**Published:** 2026-04-16

**Authors:** Mauro Feola, Federico Landra, Cosimo Angelo Greco, Roberto Lorusso, Gaetano Ruocco

**Affiliations:** 1 Cardiology Division, Regina Montis Regalis Hospital, Azienda Sanitaria Locale Cuneo 1 (ASLCN1), 12084 Mondovì, Italy; 2 Cardiology Unit, I. Veris Delli Ponti Hospital, Azienda Sanitaria Locale di Lecce, 73020 Scorrano, Italy; 3 Cardiothoracic Surgery Department, Heart and Vascular Centre, Maastricht University Medical Centre, 6202 AZ Maastrict, The Netherlands; 4 Cardiovascular Research Institute Maastricht, Maastrict University, 6202 AZ Maastricht, The Netherlands

**Keywords:** heart failure, preserved left ventricular ejection fraction, sudden cardiac death

## Abstract

Cardiac arrest is a way of demise of patients who are affected by heart failure (HF), being more frequent in those with HF with a reduced left ventricular ejection fraction (HFrEF), and is, as such, responsible for 30–50% of cardiac death. Specific data on the risk of sudden cardiac death (SCD) related to HF with a preserved ejection fraction (HFpEF) and HF with a mildly reduced ejection fraction (HFmrEF) are lacking, as well as data regarding ventricular arrhythmias in this population. Considering the 0.3% person/year incidence rate of investigator-reported ventricular tachycardia (VT) and ventricular fibrillation (VF), the rate of SCD in the analyzed population seems to be 1.3% per year. Age, gender, history of diabetes and myocardial infarction, left bundle branch block (LBBB) on electrocardiogram (ECG), and a natural logarithm of N-terminal pro B-type natriuretic peptide (NT-proBNP), identified a subgroup of HFpEF patients with a higher risk (5-year cumulative incidence of 11%) of sudden death (SD). In HFpEF patients, both glifozins and finerenone did not demonstrate a beneficial effect on SCD incidence in comparison to placebo. A significantly lower rate of SCD emerged in patients who were treated with dapaglifozin (10 vs. 26 pts) among patients with HF with an improved ejection fraction (HFimpEF), who were defined as patients with a previous left ventricular ejection fraction (LVEF) < 40%. Promising methods discussed include cardiac magnetic resonance, myocardial scintigraphy, genetic assessment, and electrophysiologic studies for predicting SCD in those patients. In conclusion, arrhythmic SCD in HFpEF patients should not be considered merely as an effect of VT/VF; bradyarrhythmia is probably more frequent and dangerous. The effects of drugs in preventing SCD in HFpEF have not been demonstrated yet.

**Key Learning Points**

*What Is Already Known*

Poor data are available regarding SCD in HFpEF and HFmrEF; there are no studies in HFmrEF and HFpEF patients designed to identify the patients’ risk, followed by individual patient treatment with or without a device.

The newly available treatment for HFpEF and HFmrEF (SGLT2is and Finerenone) did not demonstrate efficacy in SCD prevention.

The optimization of device treatment should be mandatory in HF patients at high risk.

*What This Study Adds*

SCD in HFpEF and HFmrEF is related to both VT/FV and bradyarrhythmias, which are probably more frequent and dangerous.

Different clinical and instrumental factors are related to SCD incidence in HFpEF and HFmrEF.

A more comprehensive approach, including cardiac magnetic resonance, myocardial scintigraphy, genetic assessment, and electrophysiologic studies, appears to be mandatory in order to stratify risk and to decide if patients are eligible for device implantation.

## 1. Introduction

Cardiac arrest (CA) is a way of demise of patients who are affected by heart failure (HF), and it is more frequent in HF with a reduced left ventricular ejection fraction (HFrEF), causing a percentage of cardiac deaths, ranging from 30% to 50% [1,2]. Although the role and mechanisms of sudden cardiac death (SCD) that occurs in HFrEF appear to be clarified, in patients with HF with a preserved ejection fraction (HFpEF), with a mildly reduced ejection fraction (HFmrEF), or with an improved left ventricular ejection fraction (HFimpEF), it is under debate. Moreover, when analyzing the available data in HFmrEF and HFpEF, we found different rates of both tachyarrhythmia and bradyarrhythmia [3,4,5]. Thus, to avoid SCD, identifying individual patient risk through better patient screening is mandatory.

The aim of this review is to highlight the incidence and mechanisms of SCD in patients with HFpEF and HFmrEF, as well as to examine the effectiveness of risk stratification modalities and treatment strategies in these patients. We performed our research via PubMed, evaluating randomized controlled trials, meta-analyses, original research articles, observational/retrospective, and propensity-score-matched studies from 1 January 2000 to 31 December 2025. We used the following mesh terms: “cardiac arrest” and/or “sudden death” or “sudden cardiac death” and “heart failure with preserved ejection fraction” and/or “heart failure and mildly reduced ejection fraction” and “ventricular tachycardia” and/or “ventricular fibrillation” and “bradyarrhythmias”. We considered only studies in the English language and the adult population (≥18 years old). We excluded case reports and letters.

## 2. Incidence of SCD in HFmrEF/HFpEF

The incidence of SCD was different across HF patients’ phenotypes. However, specific data on the risk of SCD related to HFpEF and HFmrEF are lacking, as well as data regarding ventricular arrhythmias in this population. In the retrospective study of Stecker et al., based on SCD in Oregon, of a total of 714 events, 22% have a mildly to moderately reduced EF (from 36% to 55%, established before demise). Only 30% of SCD cases were documented in patients with HFrEF who met criteria for prophylactic cardioverter-defibrillator (ICD) implantation [6]. Combined data, coming from four trials enrolling 13,609 patients (both HFmrEF and HFpEF), demonstrated an incidence rate of 0.3% person/year in terms of investigator-reported ventricular tachycardia (VT) and ventricular fibrillation (VF) [7]. HFmrEF patients seemed to be at a higher risk of VT/VF compared to HFpEF patients (HR 2.19). In this study, male gender, previous myocardial infarction (MI), impaired renal function, and higher plasma level of N-terminal pro B-type natriuretic peptide (NT-proBNP) are strictly related to the incidence of VT/VF. The rate of SCD in the analyzed population seemed to be 1.3% per year; however, a lack of correlation between the occurrence of VT/VF and SCD has been found, probably due to the small number of events (VT/VF) reported by the investigators [7]. This is in line with a possible low incidence of VT/VF in HFmrEF and HFpEF related to the underestimation phenomenon [8]. Indeed, VT/VF occurrence was based on the investigators’ reported rates without continuous monitoring data. Despite these findings, 25% of HFpEF and 50% of HFmrEF patients still presented with shockable rhythms after SCD. Additionally, a non-shockable rhythm, such as asystole, may indicate prior VT/VF in a considerable number of patients [9]. The VIP-HF study revealed that sustained VT occurred at a rate of 0.6 per 100 person-years, and non-sustained VT at 11.5 per 100 person-years among 113 HF patients with HFmrEF and HFpEF who received an implanted loop-recorder (ILR). The incidence of ventricular tachyarrhythmias seemed to be infrequent, while the incidence of clinically relevant bradyarrhythmias proved to be more than expected [10]. Using ambulatory cardiac monitoring, Cho et al. observed that ventricular tachycardia was more frequent in HFpEF patients compared to patients without HF (37% vs. 16%, *p* = 0.001); moreover, in the same study, an increased QTc interval was associated with a relevant risk of VT [11].

## 3. The Cause of Cardiac Sudden Death in HFpEF of HFmrEF Patients: Tachyarrhythmia or Bradyarrhythmia

The distribution of modes of death, reported by Vaduganathan et al., in HFpEF patients, obtained in three large clinical trials, revealed a SCD rate of 28%; however, only half of the SCD episodes should be considered arrhythmic (14% of total). Arrhythmias related to arrhythmic SCD include sustained VT of VF, asystole, or electromechanical dissociation [12]. In the VIP-HF study, the ILRs registered more episodes of bradycardia than expected; in about 25% of cases of death, bradycardia represented a terminal rhythm. Moreover, symptomatic bradycardia in 4.4% of patients emerged, and of these, 80% of cases required pacemaker implantation. Therefore, due to the administration of beta-blockers in 88% of the VIP-HF study cohort, impairment of bradyarrhythmia should be related to the exacerbation of conduction system disease in HFpEF patients taking this medication [10]. Therefore, Yougquist et al. investigated out-of-hospital cardiac arrest patients as a way to demonstrate the potential role of beta-blockers in SCD. They documented that patient presenting with pulseless electrical activity had 3.7 times higher therapy with beta-blockers in comparison to patients presenting with VF [13]. In the study performed by Hooks et al., initial rhythms for in-hospital cardiac arrest was VT/VF in 31.1% of cases, asystole in 9.5%, and PEA in 59.7%. VT/VF was significantly more frequent in patients with HFpEF (47.1%) and HFrEF (39.0%) compared with those without HF (22.2%; *p* < 0.01). Despite the lower reported incidence of ventricular arrhythmias in HFpEF and HFmrEF with respect to HFrEF, SCD remained one of the main contributors to mortality in these groups. While tachyarrhythmias appear more prevalent in HFmrEF, bradyarrhythmias and electromechanical dissociation are prevalent in advanced HFpEF [14]. Indeed, a recent study involving 1003 patients with hypertrophic cardiomyopathy (HCM) highlighted that primary bradycardia was present in 83 patients (both sinus node dysfunction and atrioventricular block); bradycardia was related to a higher risk of all-cause death, cardiovascular death, and HF-related death [15]. Moreover, a recent analysis in chronic heart failure (CHF) patients showed that HFpEF patients had a similar incidence of atrial fibrillation (AF) and bradyarrhythmias with respect to HFrEF, with a lower incidence of VT/VF. However, bradyarrhythmias were significantly related to all-cause death [16]. Due to the underestimation of arrhythmic events in previous studies from data being reported by investigators rather than continuous monitoring, ILR and ambulatory ECG monitoring could offer a more accurate assessment of true arrhythmic burden. Furthermore, patient-specific factors—such as age, comorbidities, renal function, and beta-blocker use—should be carefully considered when assessing SCD risk and developing personalized preventive strategies.

Therefore, the arrhythmic SCD in HFpEF patients should not be merely considered as an effect of VT/VF; bradyarrhythmias are probably more frequent and dangerous.

## 4. Predicting SCD in HFpEF

**Figure 1:**
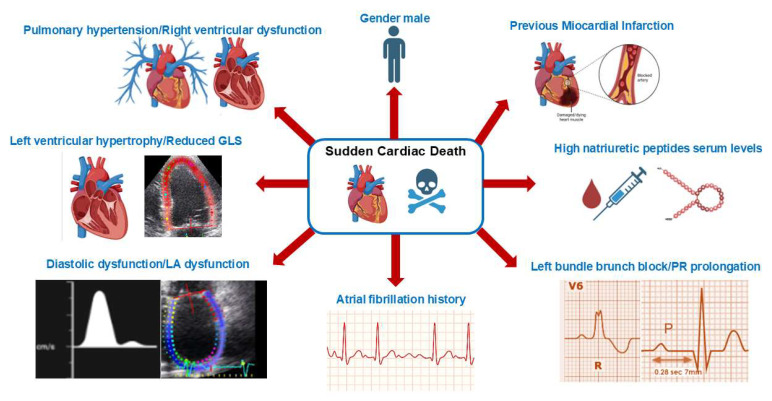
Main factors related to sudden cardiac death in heart failure patients with moderately reduced or preserved ejection fraction.

A validated risk score for predicting cardiac SCD in HFpEF patients remains a challenge. The possibility of identifying subgroups of subjects in whom the risk of SCD should be high could lead to a more aggressive attitude in terms of antiarrhythmic drugs, electrophysiological studies, and finally, an implantable cardiac defibrillator (ICD). In a post hoc analysis of the I-PRESERVE trial, published in 2014, HFpEF patients treated with irbesartan vs. placebo were categorized by using a multivariable model, including age, gender, history of diabetes and myocardial infarction, LBBB on ECG, and a natural logarithm of NT-proBNP, identifying subgroups of patients with a higher risk of SCD (5-year cumulative incidence of 11%) and those who were at low risk (4% incidence) [17]. Likewise, a post hoc analysis of the TOPCAT trial, in which HFpEF patients were treated with spironolactone vs. placebo, demonstrated that the use of six variables (age, sex, history of myocardial infarction, diabetes, presence of LBBB, and plasma level of Nt-proBNP) generated a risk score model, allowing clinicians to estimate the 5-year cumulative incidence of SCD (15.2% in the high-risk population in comparison to 2.8% in the low-risk group) [18]. In the TOPCAT trial, the rate of sudden death was about 20%, and male gender and diabetes treated with insulin were significant predictors of sudden death in HFpEF patients [19]. Inclusion in these trials of HFmrEF patients could increase the incidence of SCD with respect to HFpEF patients alone, placing doubt on the real burden of SCD in HFpEF patients [17,18,19]. Recently, Desai et al. analyzed pooled data from the DAPA-HF and DELIVER trials, showing a rate of cardiovascular mortality that ranged from 40 to 50% of deaths in HFpEF, with the largest proportion of SCD, even when the ejection fraction was greater than 60% [20]. In this sense, a prospective case-control analysis of 652 cases of SCD demonstrated a significant association of AF in SCD in patients who demonstrated a history of CHF, with significant results seen in patients with a left ventricular ejection fraction (LVEF) of both >40% and >50% [21]. This relation was confirmed in hypertensive patients with new-onset AF [22]. Moreover, echocardiography could represent a further tool for predicting SCD risk in HFmrEF and HFpEF patients. The alterations in left ventricle (LV) geometry and left ventricular hypertrophy (LVH), could be related to SCD incidence [23]. In a study performed by Aro et al., patients with a LVEF of >35%, who showed concentric remodeling, concentric hypertrophy, and eccentric hypertrophy, were more prone to SCD incidence, with a major risk demonstrated in patients with concentric hypertrophy [24]. Among echocardiographic variables, global longitudinal strain (GLS) of the LV should be used to stratify SCD risk. A sub-analysis of the TOPCAT trial showed that for every one-point increase in GLS, there was a 58% increase in the hazard of incident SCD [25]. Moreover, different studies demonstrated that lower peak left atrial (LA) strain, which represents an index of LA dysfunction, was also related to poorer outcomes in HFpEF as well as the degree of diastolic dysfunction [26,27,28]. Finally, the presence of pulmonary hypertension (PH) should be considered in HFpEF [29]. PH in HFpEF has usually been divided into isolated post-capillary PH and combined post- and pre-capillary PH, and it has been proven to be common in HFpEF patients (77–83%). Obokata et al., in serial echocardiographic controls for 4 years, demonstrated that 23% of HFpEF patients had deteriorated right ventricle function, doubling the risk of death [30]. The presence of right ventricle dysfunction (RVD) might predispose one to SCD. In an observational study conducted by the Mayo Clinic, including 5463 patients in whom a complete analysis of RVD has been performed, the presence of moderate to severe RVD significantly increased the risk of SCD (7.4% vs. 4.4% in mild RVD) [31]. Moreover, Pandat et al., in a community-based study centered on SCD, documented that RVD was associated with a significantly increased risk of SCD independently of LV function [32]. These considerations suggest future inclusion of the parameters PH or RVD in the risk assessment of SCD in patients with HFpEF [Figure 1]. Overall, the heterogeneity of the population in terms of echocardiographic features, risk factor profiles, and hemodynamic derangement did not allow for a precise SCD risk stratification of these patients. Therefore, a correct patient assessment with ECG monitoring and echocardiography could improve SCD prevention.

## 5. Influence of New HF Medications on SCD

It is widely recognized that SGLT2 inhibitors play a role in preventing SCD in HF patients.

Findings from different meta-analyses showed the protective effect of SGLT2is treatment compared to conventional medical therapy in SCD outcomes in patients affected by both diabetes with and without HF, and in patients only affected by HF [33,34,35,36,37]. The pathophysiological mechanisms involved in SCD prevention by SGLT2is are not clearly defined. Probably, different pleiotropic effects of these drugs are related to the reduction of SCD rate, such as cardiorenal protection, reduction of oxidative stress, antifibrotic effects, improvement in cardiomyocytes metabolism, and improvement in hemodynamic performance [38]. However, when analyzing SCD prevention based on HF phenotypes defined by LVEF, some differences in clinical trial evidence emerge.

In patients with HFrEF, addition of dapaglifozin to standard HF treatment led to a reduction in the rate of SCD, as confirmed by the DAPA-HF study [39].

**Figure 2:**
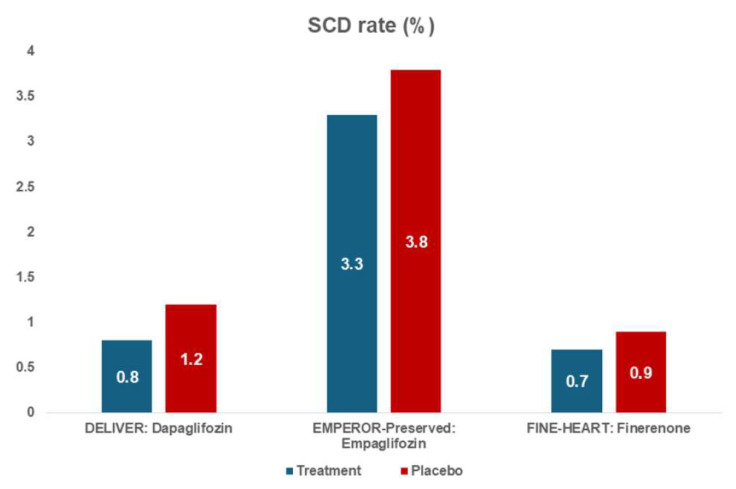
Sudden cardiac death (SCD) rate according to heart failure treatment.

However, there are no data about SCD prevention in HFrEF patients treated with empaglifozin [40]. Despite significant evidence on SCD reduction in HFrEF patients and those with diabetes treated with SGLT2is, there are not enough data in HFpEF patients. In the DELIVER trial, Vardeny et al. demonstrated a significantly lower rate of sudden death (SD) in patients treated with dapaglifozin (10 vs. 26 pts) among patients with HF with an improved ejection fraction (HFimpEF), who were defined as patients with a previous LVEF of <40% [41]. In patients with HFpEF (with an hystory of LVEF > 40%), there are no significant differences in terms of SCD rate. More precisely, when analyzing the DELIVER population, the rate of SCD was not significantly different among patients who were treated with dapaglifozin compared to patients treated with placebo (0.8% vs. 1.2%) [42]. Similarly, in the EMPEROR-PRESERVED trial, there were no differences in SCD rate in the two treatment arms (empaglifozin: 3.3% vs. placebo:3.8%) [43]. Therefore, in patients with a diagnosis of HFpEF (LVEF ≥ 40% both prior to randomization and after randomization), SGLT2is therapy seemed not to be associated with a significant reduction in SCD rate, suggesting a different pathophysiological explanation. Finerenone is a non-steroidal mineral receptor antagonist (MRA) that has recently been studied in the setting of chronic kidney disease (CKD) and diabetes mellitus (DM). The beneficial effects of this treatment in cardiovascular and HF outcomes suggested a potential role of this drug in HFpEF [44,45]. Due to this evidence, finerenone was finally studied in HFpEF patients, demonstrating a significant reduction in composite outcomes, which were worsening heart failure (WHF) events and death from cardiovascular causes [46]. Unfortunately, SCD was considered within cardiovascular death, and the clinical trials with finerenone (such as FIDELIO-DKD, FIGARO-DKD, and FINEARTS-HF) did not present its rate. However, the FINE-HEARTS analysis, which included 7008 HFpEF patients of the three clinical trials, demonstrated that the SCD rate was similar among patients who were treated with finerenone and patients treated with placebo (0.7% vs. 0.9%); the rate of SCD was mainly driven by the increase in age [47,48]. In conclusion, in HFpEF patients, both SGLT2is and finerenone did not demonstrate a beneficial effect on SCD incidence in comparison to placebo [Figure 2].

Due to this evidence, we can affirm that in HF trials involving HF patients with LVEF ≥ 40%, we do not have a beneficial effect of approved drugs on SCD incidence. This is probably due to the different phenotypes (both HFmrEF and HFpEF) of patients and the different etiology of SCD in this population (both tachyarrhythmias and bradyarrhythmias). In this sense, there was no general treatment to reduce the SCD burden. For example, beta-blockers could lead to or impair bradyarrhythmias but are effective in ventricular arrhythmias. Diuretic therapy could reduce potassium levels, favouring tachyarrhythmias. Conversely, MRA could cause hyperkalemia, leading to bradyarrhythmias. Therefore, a detailed risk assessment of SCD should be built for each patient to administer the correct therapy.

## 6. Promising Methods for Predicting SD in HFpEF

### 6.1. Cardiac Magnetic Resonance

Cardiac magnetic resonance (CMR) has reached significant attention in recent years due to its unique power for non-invasive tissue characterization [49,50]. Extensive evidence supports the use of late gadolinium enhancement (LGE) in predicting the occurrence of ventricular arrhythmias (VAs)—particularly sustained monomorphic ventricular tachycardia (SMVT)—and SCD in patients with both ischemic and non-ischemic cardiomyopathies, independently from LVEF [51,52,53,54,55,56]. Risk stratification algorithms incorporating LGE have demonstrated superior predictive performance compared to models including LVEF ≤ 35% [52,53]. Moreover, Almehmadi et al., in a cohort of patients referred for LGE-CMR and a LVEF < 55%, demonstrated that the presence of a midwall striae pattern of LGE was an independent predictor of cardiac arrest or appropriate ICD therapy with a hazard ratio (HR) of 2.4 (95% confidence interval (CI), 1.2–4.6; *p* = 0.010) [57]. This alteration was present in 30% of patients with non-ischemic cardiomyopathy and 15% of patients with ischemic cardiomyopathy. The cumulative event rate was significantly higher among those with midwall striae, particularly among those with a LVEF > 35% (40% versus 6%; *p* = 0.005).

In recent years, various post-processing techniques (primarily based on signal intensity thresholding) have been developed to enhance scar characterization and to identify arrhythmogenic substrates [54,58]. Among these, the measurement of border zone channel (BZC) mass—reflecting heterogeneous scar tissue that may harbor slow-conducting channels—has emerged as the most robust independent predictor of SMVT in ischemic cardiomyopathy, even after adjustment for baseline variables, including LVEF [54]. Of note, a cut-off of 5.15 g of BZC mass identified patients with previous VAs, with 92.4% sensitivity and 86.9% specificity (area under the ROC curve (AUC) 0.93 (0.89–0.97); *p* < 0.001). Adding BZC mass to LVEF allowed for the reclassification of 33.3% of cases (i.e., patients with prior Vas) and 39.3% of controls (net reclassification improvement = 0.73 [0.71–0.74]). Although less extensively studied, this association has also been observed in HCM [59]. Importantly, LGE post-processing seemed to have therapeutic implications, such as guiding ventricular tachycardia catheter ablation procedures [60,61,62,63,64,65].

Beyond the well-established utility of LGE, novel CMR techniques that may further enhance the detection of structural abnormalities are emerging. Among these, extracellular volume (ECV) quantification, derived from T1 mapping, enables the detection of diffuse interstitial fibrosis not identified by LGE imaging [66]. ECV maps can distinguish between normal myocardium and regions of diffuse fibrosis that could be missed by conventional techniques. Diffuse myocardial fibrosis may precede the clinical manifestation of HFpEF and is predictive of disease severity and prognosis [67]. Importantly, Schelbert et al. demonstrated that ECV is independently associated with all-cause mortality across the full spectrum of LVEF and HF stages; whereas, fibrosis quantified by LGE was not related to mortality [68]. Other studies in this setting identified ECV as a strong predictor of adverse outcomes in HFpEF, including HF hospitalizations and death [69,70]. Additionally, Brown et al. demonstrated that patients with HFmrEF share phenotypic features with both HFpEF (in terms of diffuse myocardial fibrosis) and HFrEF (in relation to occult ischemic heart disease) [71]. More recently, Di Marco et al. reported that in patients with non-ischemic cardiomyopathy (median LVEF 42%, IQR 32–48), an ECV ≥ 30%, in combination with LVEF ≤ 35% and LGE presence, provided excellent predictive value for VAs and sudden death (SD), yielding a Harrell’s C-statistic of 0.82 [72]. Patients positive for all three criteria exhibited an annual arrhythmic event rate of 7.5%, compared to just 0.2% in the low-risk group (defined as any LVEF and either both LGE-negative and ECV < 30%, or only one positive marker). ECV ≥ 30% was the strongest independent predictor of arrhythmic events after adjustment for LGE and LVEF (HR 14.1, *p* = 0.010), demonstrating a sensitivity of 91%, specificity of 70%, negative predictive value of 99.8%, and positive predictive value of 5%, the latter being influenced by the overall low incidence (2%) of the primary outcome in the studied cohort of 691 patients. In contrast, neither native T1 values nor global longitudinal strain assessed by CMR were associated with the composite primary outcome, which included appropriate ICD therapies, sustained VAs, resuscitated cardiac arrest, and SD [72].

A promising field of research is the application of novel artificial intelligence and deep learning approaches to further refine arrhythmic risk stratification [73,74,75]. A deep learning process blending neural networks and survival analysis has been proposed to predict patient-specific survival curves directly from raw contrast-enhanced CMR images in ischemic cardiomyopathy patients, therefore avoiding manual threshold-based segmentations [76]. Also, digital twin technologies, namely digital representations of organs capable of simulating personal health conditions and predicting disease trajectories, are being developed to specifically address the issue of SCD prediction. A small study conducted by Deng et al. demonstrated that a virtual heart electrophysiological study (EPS) simulation successfully predicted arrhythmic risk in all included myocardial infarction patients with an LVEF > 35% [77]. Another study proposed a digital twin (DT) model not only incorporating CMR data but also information derived from computed tomography scans regarding myocardial fat infiltration, which has been demonstrated to be a primary driver of substrate arrhythmogenicity [78,79,80]. On the contrary, O’Hara et al. proposed a DT model using both LGE and T1-mapping CMR-derived data in a cohort of HCM, successfully predicting VAs risk [81]. Therefore, the arrhythmic risk stratification of HFpEF and HFmrEF patients should be evaluated, including CMR variables. The addition of LGE, BZC, and ECV to LVEF and clinical and hemodynamic factors could increase the capacity to detect patients at high SCD risk to optimize both medical and device therapy.

### 6.2. Myocardial Scintigraphy (SPECT)

^123^I-meta-iodobenzylguanidine (^123^I-mIBG) is a norepinephrine analog used in SPECT imaging to assess the functional integrity of cardiac sympathetic nerve terminals. In patients with HF, this imaging modality may offer important prognostic information; the abnormalities in cardiac sympathetic innervation have been associated with the development of malignant VAs and SCD. Denervation of viable myocardium may lead to heterogeneous sympathetic stimulation responses, creating a favorable electrophysiological substrate to both ventricular tachycardia (VT) and ventricular fibrillation (VF). Calkins et al. used C^11^-HED PET in patients with sustained VT, demonstrating a relationship between areas of denervation and ventricular refractoriness [82].

^123^I-mIBG imaging has been investigated as a tool to predict the inducibility of VAs. In a study by Bax et al., only the extent of the ^123^I-mIBG defect on late SPECT imaging was associated with inducible VAs during EPS [83]. Arora et al. reported that patients experiencing ICD appropriate shocks exhibited significantly lower heart-to-mediastinum (H/M) ratios, higher defect scores, and more widespread denervation [84]. Similarly, Boogers et al. found that sympathetic denervation on MIBG imaging predicted both appropriate ICD therapy and the composite outcome of ICD intervention or cardiac death [85]. Specifically, patients with a summed defect score > 31 had a 75% sensitivity and 82% specificity for experiencing potentially lethal arrhythmic events requiring ICD treatment. Multiple studies have shown that the H/M ratio on planar ^123^I-mIBG imaging correlates with adverse outcomes in both ischemic and non-ischemic HF [86,87,88]. In two analyses, the H/M ratio outperformed LVEF in outcome prediction [89,90]. However, most deaths in those studies were due to HF progression rather than SCD, and data specifically linking the H/M ratio to SCD risk remain limited. Different studies demonstrated that a reduced H/M ratio predicted SCD in patients with mild-to-moderate HF [91,92]. In a multivariate analysis, both washout rate (WR) and LVEF emerged as independent predictors of SCD, but WR offered superior specificity and predictive accuracy. Importantly, WR retained its prognostic utility, even in patients with LVEF > 35%, suggesting that MIBG imaging may identify candidates for ICD implantation at risk for SD who would not otherwise meet current guideline criteria [92]. The ADMIRE-HF showed that patients with an H/M ratio ≥ 1.60 experienced a 60% reduction in the incidence of major cardiac events—including death, HF progression, and VAs—compared to those with an H/M < 1.60. Additionally, patients with lower H/M ratios had over a five-fold increase in 2-year all-cause mortality (16.1% vs. 3%). The H/M ratio provided prognostic information incremental to that of LVEF and BNP, both independently and in combination [93]. The H/M ratio was related to an increase in arrhythmia burden. This is probably due to the consequence of regional mismatches between perfusion and innervation, together with sympathetic dysfunction, which increase heart susceptibility to arrhythmias [94].

Despite these encouraging results, no definitive data have yet confirmed how this global measurement of cardiac sympathetic innervation might predict spontaneous VT occurrence or inducibility during EPS with a high specificity. Recently, Seo et al. investigated whether ^123^I-mIBG SPECT imaging could help predict outcomes in patients with HFpEF [95]. While this imaging method is known to have prognostic value in patients with HFrEF, its role in HFpEF was previously unclear. A total of 148 patients with non-ischemic HFpEF, who underwent ^123^I-MIBG imaging, were followed after an episode of acute decompensated HF. The authors calculated a total defect score (TDS) using a semiquantitative measure (considering a 0 to 4 uptake score from normal to total perfusion defect for each of the 17 left ventricular segments). Over an average follow-up of 2.4 years, patients with higher TDS values had significantly more cardiac events, such as HF hospitalization or cardiac death (primary outcome). Notably, 49 patients died, including 23 cardiac deaths (of which eight had been labeled as SCD) and 26 non-cardiac deaths [95]. The TDS proved to be a stronger predictor of the primary outcome than the late H/M ratio and WR [95]. These findings suggest that ^123^I-MIBG SPECT imaging offers meaningful prognostic information in HFpEF and could be a valuable tool for risk assessment in this patient population. MIBG imaging could help refine current ICD implantation criteria—identifying high-risk patients who fall outside standard guideline recommendations.

### 6.3. Genetic Assessment

Genetic markers offer a promising avenue for improving risk stratification among the large subset of patients who do not meet current guideline-directed indications for ICD therapy but who remain vulnerable to SCD. These markers are now accessible on a broad scale and at relatively low cost. In a pivotal study, Sandhu et al. evaluated the utility of a validated genome-wide polygenic score for coronary artery disease (GPS_CAD_) for stratifying the risk of sudden arrhythmic death (SAD) in an intermediate-risk cohort with established coronary artery disease (CAD) but without severe systolic dysfunction or a primary prevention ICD indication [96,97,98]. The GPS_CAD_, comprising over 6 million common genetic variants, had already been shown to predict incident CAD, and was assessed in this context for its association with SAD and competing causes of mortality over an 8-year follow-up period. Within the PRE-DETERMINE study cohort, patients in the highest decile of GPS_CAD_ exhibited a 77% increased risk of SAD after adjustment for LVEF, clinical variables, and ECG findings [98]. Notably, GPS_CAD_ was not associated with non-sudden cardiac deaths, supporting its specificity as a predictive marker for SAD. The authors concluded that their findings substantiate the hypothesis that genetic predisposition plays a significant role in the risk of SAD among patients with CAD [98].

In patients with non-ischemic cardiomyopathy (NICM), expert consensus guidelines recommend genetic testing, particularly in the presence of a suggestive family history [99]. Genetic testing in NICM can identify individuals at an elevated risk for VAs and guide decisions regarding primary prevention ICD implantation. Rare pathogenic variants in genes such as *LMNA*, *DSP*, *PLN*, *FLNC*, and *RBM20* have been associated with increased VAs risk, often in the setting of HFmrEF or even HFpEF [100,101,102,103]. The 2017 ACC/AHA/HRS guidelines on ventricular arrhythmias provided specific recommendations for ICD implantation in *LMNA*-related NICM, including criteria such as LVEF < 45%, male sex, and non-sustained ventricular tachycardia (NSVT) [104]. Similarly, the 2022 ESC guidelines for the management of ventricular arrhythmias and the prevention of SCD gave a class of evidence IIa for ICD implantation in patients with dilated or hypokinetic non-dilated cardiomyopathy (DCM/HNDCM) and *LMNA* variants who present a ≥10% estimated 5-year risk of VA and either LVEF < 50%, NSVT, or atrioventricular conduction delay [105]. Furthermore, a class IIa indication is extended to DCM/HNDCM patients with a LVEF between 36–50% and at least two additional risk factors, including unexplained syncope, pathogenic variants in *PLN*, *FLNC*, or *RBM20*, LGE on CMR, or inducible SMVT during EPS [105]. Although risk stratification for VAs in NICM linked to *DSP*, *PLN*, *FLNC*, and *RBM20* is still evolving, evidence suggests that a substantial proportion of VAs events in these populations occur despite preserved systolic function (LVEF > 45%), highlighting the importance of gene-specific risk models.

### 6.4. Invasive Electrophysiological Study

Beyond the abovementioned stratification tools, additional predictors of SCD have been proposed. In post-infarction patients with preserved or mildly reduced LVEF, there are no data supporting the implantation of a primary prophylactic ICD. These patients constitute a heterogeneous group in terms of arrhythmic substrate, and efforts are ongoing to identify those at the highest risk of SCD. The role of EPS has been established by the MUSTT trial in patients with CAD, reduced LVEF (≤40%), and asymptomatic NSVT, in whom VT/VF inducibility during EPS proved to identify patients who may benefit from an ICD [106]. In the PRESERVE-EF study, among 575 post-infarct patients with a LVEF ≥ 40% and at least one non-invasive ECG risk factor assessed more than 40 days after myocardial infarction, 41 were inducible for VT/VF during EPS and received an ICD [107]. During the 32-month follow-up, no SCD occurred, and nine of the thirty-seven patients with ICDs received appropriate therapies [107]. However, it remains uncertain whether appropriate ICD interventions can be reliably considered a surrogate for SCD in patients with preserved LVEF, and randomized trials are needed. Finally, EPS is recommended in post-infarct patients when syncope remains unexplained after a non-invasive evaluation to guide clinical management [108].

In the context of DCM/HNDCM, as previously mentioned, inducibility of SMVT during EPS is recognized as a risk factor for SD in patients with a LVEF of between 36–50%, particularly if associated with at least one additional risk factor, such as unexplained syncope, pathogenic variants in PLN, FLNC, or RBM20, or the presence of LGE on CMR [108]. Nonetheless, prospective studies evaluating the clinical benefit of ICDs in patients with DCM/HNDCM and intermediate systolic dysfunction but with such risk factors are currently lacking. As in CAD, unexplained syncope in these patients warrants further evaluation, and EPS may help clarify the underlying mechanism. Indeed, data in DCM patients with mildly reduced EF suggest an added value of EPS: in patients with a LVEF ≥ 40% and unexplained syncope who underwent ICD implantation following a positive EPS, 80% experienced appropriate ICD therapy during follow-up, while no SCD or symptomatic VAs were reported in non-inducible patients [109].

The prognostic value of programmed ventricular stimulation (PVS) in patients with mildly reduced or preserved LVEF and myocardial scarring has been recently investigated by Soris et al., considering both ischemic and non-ischemic etiologies (52% and 48%, respectively) [110]. Indications for PVS were mostly NSVT (45%) and/or syncope (38%). The primary endpoint was the occurrence of a MAE, namely SCD, VT/VF, and appropriate ICD therapy. In the 168 included patients, the mean LVEF was 54% ± 9%, while 46% had a normal LVEF (>55%). Inducibility during PVS was observed in 21 patients (13%). Inducible patients had significantly lower LVEFs (48% ± 5% vs. 55% ± 9%, *p* < 0.001) and were more frequently ischemic (*p* = 0.09) [110]. Over a mean follow-up of 46 ± 38 months, a MAE occurred in 43% patients with positive PVS vs. 2.7% with negative PVS. Inducibility during PVS provided high rule-out performance, with a 97% negative predictive value for the prediction of MAE, and a fair rule-in performance, with a 43% positive predictive value (sensitivity 69%; specificity 92%). The authors concluded that PVS may be a useful tool to discriminate patients with myocardial scars and a LVEF of ≥40% at increased arrhythmic risk [110]. Apart from syncope, NSVT could represent a valuable new indication for PVS. Therefore, this study filled a gap in the literature regarding arrhythmic risk stratification of patients with LVEF > 40%, particularly for patients without syncope. It confirmed the results of the PRESERVE-EF study, extending them to more easily applicable criteria, and extended the findings of the MUSTT trial to patients with more preserved LVEF.

Patients presenting with hemodynamically well-tolerated, spontaneous SMVT have historically been excluded from secondary prevention trials. Consequently, strategies aimed at reducing the risk of SCD in this population must be classified as primary prevention. This clinical scenario most commonly occurs in individuals with preserved or mildly reduced LVEF, where relatively intact systolic function may allow for adequate cardiac output during VT episodes. The acute life-threatening potential of these arrhythmias depends on several factors, including VT cycle length, baseline cardiac function, and the clinical context.

ICDs have frequently been used in this population, despite the lack of demonstrated survival benefit in secondary prevention trials involving patients with LVEF ≥ 35% [111]. This highlights the ongoing debate over whether appropriate ICD therapies can reliably serve as a surrogate marker for SCD in patients with non-reduced LVEF. Currently, there is insufficient evidence to definitively support a survival benefit from ICD implantation in patients with HFmrEF or HFpEF who present with spontaneous, hemodynamically well-tolerated, or inducible SMVT. Given that even in a secondary prevention context, no survival benefit has been demonstrated in patients with LVEF > 35%, the expected benefit in primary prevention for hemodynamically tolerated SMVT is likely to be even lower [111].

In selected post-myocardial infarction patients with preserved or mildly reduced LVEF, catheter ablation has emerged as a therapeutic alternative for managing well-tolerated VT, even in the absence of ICD backup. A small, single-center retrospective study evaluated patients with CAD, LVEF > 40%, and hemodynamically tolerated VT who underwent catheter ablation as first-line therapy (mean LVEF 48 ± 6%) [112]. The procedure successfully eliminated 90% of clinical and 58% of inducible VTs. If sustained VT remained inducible post-ablation, an ICD was implanted (in 42% of cases). Over a mean follow-up of 3.8 years, overall mortality was 42%, independent of ICD implantation (*p* = 0.47), and no SD occurred among patients who did not receive an ICD. VT recurrences were observed in 11% of those in whom all inducible VTs had been successfully eliminated.

A larger multicenter European retrospective study by Maury et al. evaluated 166 patients with a LVEF of >30% and well-tolerated SMVT treated with catheter ablation alone, comparing their outcomes to a control group of 378 patients who received an ICD [113]. Among the ablation group, 55% had CAD, and the mean LVEF was 50%. After a mean follow-up of 32 months, overall mortality was similar between groups (12%), with only four cases of SD (2.4%). Acute ablation outcomes have been reported to be similar between CAD and DCM [114,115]. However, VT recurrence rates are usually higher in DCM patients (VT-free survival 40.5% in DCM vs. 57% in CAD at 1 year) [115].

These findings suggest that both ICD implantation and catheter ablation may be viable options for patients with preserved or mildly reduced LVEF who present with hemodynamically tolerated SMVT, even though their effective impact on SCD reduction remains unclear. The ongoing International Multicenter Project Comparing Radiofrequency Ablation Versus Implantable Defibrillator After Well-tolerated Ventricular Tachycardia in Ischemic Heart Disease with Minimally Impaired Ejection Fraction (VIVA trial; NCT06294028) aims to test, within a randomized controlled trial design, whether catheter ablation may be superior to ICD implantation in patients with CAD and a LVEF of >35% who experience hemodynamically well-tolerated SMVT—based on retrospective data suggesting comparably low rates of SD in this population [116].

**Figure 3:**
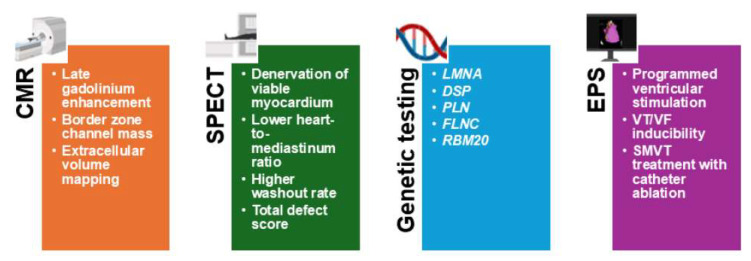
Promising tools for sudden cardiac death risk stratification.

EPS inducibility is considered a surrogate of spontaneous SMVT and, therefore, suffers from the same limitations regarding its capability of predicting malignant VAs in patients with non-reduced LVEF. Given the heterogeneous and sometimes limited life-threatening potential of SMVT in these patients, it remains uncertain whether ICD therapy, catheter ablation, or pharmacological treatment alone is sufficient to mitigate the actual risk of SCD. Appropriate ICD therapies, considered as a whole and not distinguishing between types of therapy (anti-tachycardia pacing or shocks) and VT cycles, are probably an imperfect proxy of SCD. A more refined risk stratification approach and outcome definition are therefore needed to identify which patients truly benefit from invasive interventions, and to avoid overtreatment in those at lower risk. Until more robust evidence emerges, treatment decisions should be individualized, balancing arrhythmic risk, comorbidities, patient preferences, and procedural risks [Figure 3].

## 7. Conclusions

SCD showed a different rate across HFmrEF and HFpEF patients’studies. The incidence was various, ranging from 0.5 to 30%. Moreover, both tachyarrhythmias and bradyarrhythmias were related to SCD events [107]. However, among different guideline documents, there were no specific indications for device implantation in these patients due to a lack of large, randomized data in HFmrEF and HFpEF patients [105,117,118,119]. Indeed, without a specific diagnosis of bradyarrhythmias or tachyarrhythmias, it is necessary to have a precise screening program to identify high-risk patients. In this setting of patients, known factors related to SCD are male gender, previous MI, diabetes mellitus, left bundle branch block, long PR, left ventricle hypertrophy, lower left ventricle and left atrial longitudinal strain, and PH/RVD presence. New specific parameters from alternative diagnostic tools, such as CMR, SPECT, genetic testing, and EPS, are needed to better stratify SCD in HFmrEF and HFpEF patients. Further research will be mandatory to better stratify HFmrEF and HFpEF patients, building a predictive model that can steer clinicians in both medical and device treatment for these patients.

## Data Availability

No new data were generated or analyzed in support of this research.

## Abbreviations

HF: heart failure; HFrEF, heart failure with reduced ejection fraction; HFmrEF, heart failure with mildly reduced ejection fraction; EF, ejection fraction; SCD, sudden cardiac death; SD, sudden death; ICD, implantable cardioverter defibrillator; PEA, pulseless electrical activity; VT, ventricular tachycardia; VF, ventricular fibrillation; LA, Left atrial; RVD, Right ventricular dysfunction; PH, Pulmonary hypertension; GLS, Global longitudinal strain; LV, Left ventricle; LVH Left ventricular hypertrophy; LVEF, Left ventricular ejection fraction; SGLT2i, sodium-glucose cotransporter-2 inhibitor; MRA, mineral receptor antagonist; CKD, chronic kidney disease; DM, diabetes mellitus; CMR, cardiac magnetic resonance; LGE, late gadolinium enhancement; VA, ventricular arrhythmia; SMVT, sustained monomorphic ventricular tachycardia; BZC, border zone channel; HCM, hypertrophic cardiomyopathy; DCM, dilated cardiomyopathy; ACM, arrhythmogenic cardiomyopathy; ECV, extracellular volume; ^123^I-mIBG, ^123^I-meta-iodobenzylguanidine; EPS, electrophysiological study; WR, washout rate; TDS, total defect score; SAD, sudden arrhythmic death; CAD, coronary artery disease; NICM, non-ischemic cardiomyopathy; NSVT, non-sustained ventricular tachycardia; HNDCM, hypokinetic non dilated cardiomyopathy.
